# Detecting Transitions from Stability to Instability in Robotic Grasping Based on Tactile Perception

**DOI:** 10.3390/s24155080

**Published:** 2024-08-05

**Authors:** Zhou Zhao, Dongyuan Zheng, Lu Chen

**Affiliations:** 1 School of Computer Science, Central China Normal University, Wuhan 430079, China; zhaozhou@ccnu.edu.cn (Z.Z.); zhengdongyuan@mails.ccnu.edu.cn (D.Z.); 2 Hubei Engineering Research Center for Intelligent Detection and Identification of Complex Parts, Wuhan 430079, China; 3 Institute of Big Data Science and Industry, School of Computer and Information Technology, Shanxi University, Taiyuan 030006, China

**Keywords:** tactile sensor, robotic grasping, grasp stability prediction

## Abstract

Robots execute diverse load operations, including carrying, lifting, tilting, and moving objects, involving load changes or transfers. This dynamic process can result in the shift of interactive operations from stability to instability. In this paper, we respond to these dynamic changes by utilizing tactile images captured from tactile sensors during interactions, conducting a study on the dynamic stability and instability in operations, and propose a real-time dynamic state sensing network by integrating convolutional neural networks (CNNs) for spatial feature extraction and long short-term memory (LSTM) networks to capture temporal information. We collect a dataset capturing the entire transition from stable to unstable states during interaction. Employing a sliding window, we sample consecutive frames from the collected dataset and feed them into the network for the state change predictions of robots. The network achieves both real-time temporal sequence prediction at 31.84 ms per inference step and an average classification accuracy of 98.90%. Our experiments demonstrate the network’s robustness, maintaining high accuracy even with previously unseen objects.

## 1. Introduction

Given the increasing application of robotics across diverse domains, the demand for efficient and stable interactions with unknown environments and humans has risen significantly [1]. However, these interactions are often unstable, particularly when tasks involve tools or when interacting with dynamic environments. Unlike humans, who effortlessly adapt to such situations, robots generally lack these adaptive capabilities and have not been traditionally designed to handle unstable interactions [2,3].

**Figure 1:**
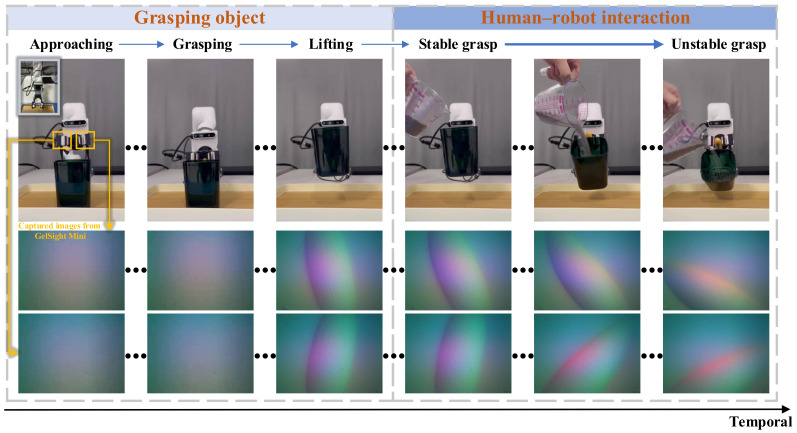
An example of a robot grasping, shifting from a stable grasp to an unstable grasp as the mass of the grasped object increases. In the diagram, the grasping process is divided into two phases: (1) Approaching the object, grasping it, and lifting it; (2) Incrementing the gripper’s load, resulting in the gradual descent of the cup. The shift from a stable to an unstable grasp is visually captured through images recorded by the tactile sensor GelSight.

As shown in Figure 1, when a robot grasps an object like a cup, the initial grasping state is stable. However, during human–robot interaction, the stability of the robot’s grip on the cup can undergo a transition from a stable to an unstable state. For example, if a person pours liquid into the cup, increasing its weight, it disrupts the robot’s previously stable grasp. The dynamic change in weight significantly influences the stability of the robot’s grip. Providing timely feedback to the human during such interactions is crucial. This immediate feedback not only enhances the overall human–robot interaction, but also assists in maintaining a coherent and safe collaboration. Keeping the human informed about the changing dynamics of the grasping state allows for better coordination and adjustment, contributing to a smoother and more effective interaction between the human and the robot. Recent advancements in robotic manipulation [4,5] emphasize the need for adaptive grasping strategies that can respond to changing conditions in real time. This provides a strong rationale for our focus on detecting transitions from stability to instability, especially in dynamic human–robot interaction scenarios.

However, in the field of robotic manipulation, most current research focuses on strategies to achieve stable grasps on arbitrary objects, promptly identify grasp failures, and on implementing preventive measures to avoid such failures [6], which overlooks the potential changes in the manipulation state that may occur due to human involvement after the initial stable manipulation. While significant progress has been made in slip detection and control algorithms for object manipulation [7,8], there remains a gap in addressing the dynamic nature of human–robot interaction, particularly in scenarios wherein the object’s properties change during manipulation. This limitation is particularly evident in collaborative tasks wherein humans and robots interact closely, potentially altering the conditions of the manipulated object. Some researchers, exemplified by Yang et al. [2] and Lu et al. [9], have ventured into addressing this issue, focusing on the challenge of instability in human–robot interaction from the perspective of robot controller development, and the quest for effective solutions is still ongoing. Subsequent efforts by Rubert et al. [10] involve the utilization of mathematical and physical models encompassing geometry, kinematics, and dynamics to calculate stable grasps, but these models face challenges in transferring seamlessly to the real world, encountering difficulties in accurately representing physical interactions between a manipulator and an object. Additionally, Fang et al. [11] introduced an innovative approach by utilizing visual information, presenting a visual-guided robotic system specifically engineered for achieving stable object grasping. It is noteworthy that prevailing solutions to instability are often predefined, incorporating methods such as direct visual observation or the consideration of specific variables, like trajectory [12], to tackle this challenge.

**Figure 2:**
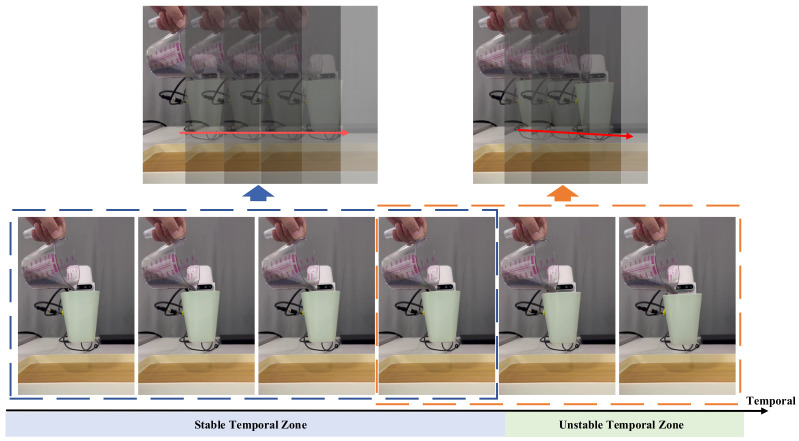
Description of stable and unstable temporal zone. During the human–robot interaction in Figure 1, when a robot transitions from a stable to an unstable grasping state, the temporal region between the stable state and the vicinity of the stability threshold is referred to as the stable temporal zone. Beyond this threshold, slipping occurs, marking the entry into an unstable state.

With the continuous advancement of tactile sensors [13,14,15], like GelSight [16], DIGIT [17], TacTip family [18], DenseTact [19], and GelFinger [20], the trend of detecting manipulation stability based on tactile information is gaining momentum. For example, Chen et al. [21] provided a comprehensive overview of tactile sensors for friction estimation and incipient slip detection, highlighting the diversity in sensor technologies, including capacitive, piezoelectric, and optical sensors. Similarly, the work by Wang et al. [22] discussed the application of the PapillArray optical tactile sensor for incipient slip detection, demonstrating the effectiveness of learning-based methods for enhancing robotic gripping performance. James et al. [23] engineered a biomimetic optical tactile sensor for rapid slip detection. Veiga et al. [24] introduced a novel slip prediction method to achieve stable object manipulation, and Calandra et al. [25] monitored incipient slip to achieve stable grasps. Informed by comprehensive surveys and case studies in diverse robotic environments [26,27], we aim to explicitly address the need for robust, real-time stability detection in various settings, including those involving human–robot collaboration. However, these studies primarily focus on detecting grasping stability during the lifting phase (see the phase of grasping object in Figure 1), rather than within the phase of human–robot interaction. Therefore, in this paper, we comprehensively address the impact of grasping position, applied force, and fluctuations in the object’s weight on grasping stability during human–robot interaction. Our primary objective is to provide corrective reminders for humans when the robot shifts from stable manipulation to an unstable state based on tactile sensing, enabling humans to conclude the interaction. The primary contributions of this paper include the following:

(1) Division of stable/unstable temporal zones. As shown in Figure 2, we explicitly introduce the stable/unstable critical point, demarcating the boundary between the stable and unstable temporal zones. Unlike other methods that primarily focus on adjusting the stability of the grasping process, we recognizes the grasping state transitions from stable to unstable due to external disturbances, even when it is initially in a stable state.
(2) Spatio-temporal information. The dynamics of human–robot interaction are intricate, and relying on a single frame for state change prediction is suboptimal. Therefore, we employ a sliding window to sample consecutive frames, harnessing temporal information to enhance prediction accuracy.
(3) Stable/unstable prediction. We propose a real-time dynamic state sensing network tailored for predicting changes in the robot’s state through analysis of a tactile sensing dataset. This model provides instantaneous feedback to humans during human–robot interaction, thereby improving the overall smoothness and effectiveness of collaboration between humans and robots. The network achieves both real-time temporal sequence prediction, with an inference step duration of 31.84 ms, and an impressive average classification accuracy of 98.90%.

## 2. Related Work

Tactile sensing plays a crucial role in robotic manipulation [28]. Traditional tactile sensors measure the deformation of surfaces under pressure to obtain tactile information during interaction [29]. However, with the advancement of new materials, an array of novel tactile sensors has been designed, expanding the application in robot manipulation tasks. This includes soft visual-based tactile sensors that mimic human skin, providing tactile sensing capabilities closely resembling those of humans. Simultaneously, the rapid development of deep learning has made visual-based tactile sensors combined with deep learning methods increasingly popular [30]. This integration facilitates smoother and more effective interactions between humans and robots. Hence, we will introduce some previous works on visual-based tactile sensors and deep learning methods in human–robot interaction, respectively.

### 2.1. Visual-Based Tactile Sensors

In visual-based tactile sensors, images of the deforming sensing surface are captured to extract tactile features [31]. Typically, this soft sensing surface is fitted with markers or pins on its inner side, and the camera records the displacements of these markers or pins [32,33]. Alternatively, some sensors detect the imprints left by external objects on the sensing skin [34,35,36]. This approach requires a larger sensor form factor to house the camera, its lighting, and to maintain the necessary distance from the sensing surface for an optimal view.

Van Duong et al. [37] introduced TacLINK, a large-scale tactile sensing system designed for robotic links. TacLINK can be assembled into a complete tactile sensing robot arm, offering scalability in size, durability, and cost-effectiveness, while delivering high performance. This versatility makes it suitable for designing robotic arms, prosthetic limbs, humanoid robots, and more. Xu et al. [38] presented a prototype that captures both visual and tactile data through a fusion of vision and tactile information, aimed at assessing the overall quality of flexible materials. Kara et al. [39] developed a vision-based surface tactile sensor to characterize and identify the sensitivity required for the reliable detection of polyps. Lin et al. [40] proposed GelSplitter, a novel framework featuring a multi-modal visual tactile sensor with synchronized multi-modal cameras, designed to mimic a more human-like tactile receptor.

### 2.2. Visual-Based Tactile Sensors in Human-Robot Interaction

Visual-based tactile sensors serve a dual purpose: they not only offer tactile feedback to enhance robotic manipulation capabilities but also provide tactile information, such as sensing the texture of objects, to convey a human-like sense of touch [41]. This additional information enhances communication between humans and robots, enabling humans to make informed and reasonable actions during human–robot interaction.

During human–robot interaction, Huang et al. [42] presented a robotic system equipped with a fully soft and inherently safe tactile interface. This interface, sized appropriately for interaction with human upper limbs, delivered detailed tactile sensory data via depth camera imaging of the soft interface. This innovative design empowered the robot to react to pokes from a human finger, adjusting its pose in response to tactile input. Agarwal et al. [43] pioneered the development of the first comprehensive optical tactile simulation system for a GelSight [16] sensor. This system, utilizing physics-based rendering techniques, delivered high-resolution, compact, and cost-effective data. It proved instrumental for achieving precise in-hand manipulation and facilitating human–robot interaction. Andrussow et al. [44] presented a pioneering soft vision-based tactile sensor named Minsight, designed to emulate the size and shape of a human fingertip. This sensor was used to generate high-resolution maps of 3D contact force by combining deep learning methods. The experimental results underscored Minsight’s ability to furnish robots with detailed fingertip touch sensing, a crucial element for achieving dexterous manipulation and facilitating physical human–robot interaction.

However, the previously mentioned visual-based tactile sensors primarily emphasize providing high-resolution tactile information, overlooking considerations for the robot’s state change. In this paper, we specifically address the transition of the robot’s state from an initial stable state to an unstable state based on tactile information gathered from tactile sensors during human–robot interaction, which is for the development of a new method for detecting slippage. This approach aims to alert humans to anticipate changes, promoting more informed and deliberate interactions.

### 2.3. Visual-Based Tactile Sensors with Deep Learning

With the rapid development of deep learning, an increasing number of researchers are exploring the integration of deep learning methods with visual-based tactile sensors. The goal is to deliver real-time sensing information and appropriate interaction methods between humans and robots. Substantial evidence suggests that leveraging deep learning methods can significantly enhance the performance of human–robot interactions [45,46]. To date, the majority of deep learning methods are constructed upon foundational architectures rooted in convolutional networks, with notable examples including VGG [47], ResNet [48], and DenseNet [49], etc.

Deep learning methods applied in the field of human–robot interaction signify additional effective applications built upon foundational network architectures. For example, Ding et al. [50] employed the TacTip [18] optical tactile sensor and trained a neural network to predict the locations and angles of edges when in contact with the sensor. Sferrazza et al. [33] designed a visual-based tactile sensor and employed an artificial deep neural network to execute tactile sensing tasks with high accuracy, particularly for a specific indenter. The sensor exhibited spatial resolution and sensing range comparable to the human fingertip. Subsequently, he extended the work by reconstructing the distribution of three-dimensional contact forces. This was achieved through training a customized deep neural network entirely on simulation data, showcasing promising generalization capabilities to previously unseen contact conditions [51]. Takahashi et al. [52] presented a deep neural network that estimates tactile properties, such as slipperiness or roughness, solely from visual perception. This model extended an encoder–decoder network, with the latent variables encompassing both visual and tactile features. The outcomes of these works serve as compelling evidence showcasing the effectiveness of incorporating deep learning methodologies in the field of robotic manipulation.

However, our method distinguishes itself from previous methods in two key aspects. Firstly, we comprehensively leverage spatial–temporal information by utilizing consecutive frame samples from a video as inputs to the classification model. This ensures that the model’s classification accuracy is not solely reliant on a single frame, enhancing its robustness to temporal dynamics. Secondly, we employ the convolutional neural network (CNN) [53] framework to extract spatial features. The features extracted by the CNN are then fed into a sequential model such as long short-term memory (LSTM) [54] for temporal processing. In time series forecasting, the sequential model is typically employed to capture long-term dependencies in the data. This enables these components to adapt their internal states based on different segments of the time series, allowing for the retention and omission of specific information. Ultimately, this method facilitates real-time feedback on the robot’s state changes, providing adaptability to dynamic scenarios.

## 3. Preliminary Work

### 3.1. Robotic Platform

**Figure 3:**
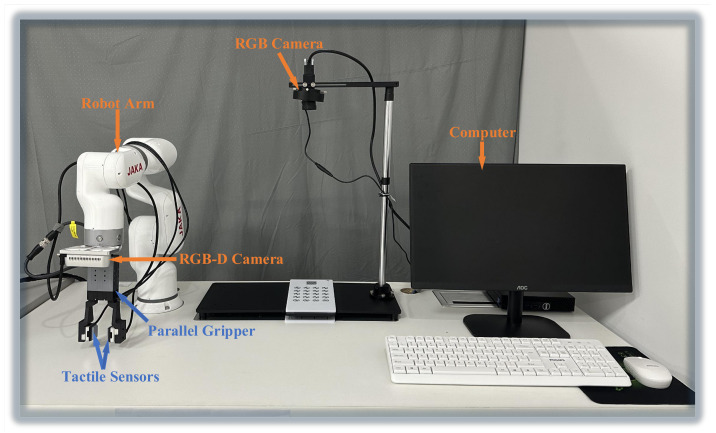
Robotic platform. It includes a 6-axis robot arm (JAKA MiniCobo by JAKA Robotics), a two-jaw parallel gripper (PGE-50-26 by DH-Robotics), a RGB-D camera (Intel Realsense D435i; Intel Corporation, Santa Clara, CA, USA), a computer based on LINUX (Ubuntu 20.04.6 LTS), and two tactile sensors (GelSight Mini).

As shown in Figure 3, we set up a six degrees-of-freedom (DOF) robot arm, manufactured by JAKA Robotics (Simpang Ampat, Malaysia) and referred to as JAKA MiniCobo. At the end of the robot arm, we fix a two-jaw parallel gripper (PGE-50-26 by DH-Robotics (Shenzhen, China)) for grasping tasks. We then replace original gripper fingers with 3D printed fingers made of polylactic acid (PLA) material, facilitating the integration of tactile sensors. The tactile sensors (GelSight Mini) is produced by GelSight (Waltham, MA, USA), which is a soft, high-resolution tactile sensor that mimics human skin to sense the shape of an object on contact, accurately capturing the surface topography (see Figure 1). These tactile sensors are seamlessly connected to a computer, transmitting captured images for further analysis and processing.

### 3.2. Data Collection from Tactile Sensors

We collect tactile data during human–robot interaction, starting from the time when the robot completes stable grasp (see Figure 1). Numerous factors influence grasp stability, including the position at which an object is grasped, the applied grasping force, and fluctuations in the object’s weight. Consequently, we establish varying levels of grasping force and diverse grasp positions for the same object, while also accounting for fluctuations in the object’s weight.

**Figure 4:**
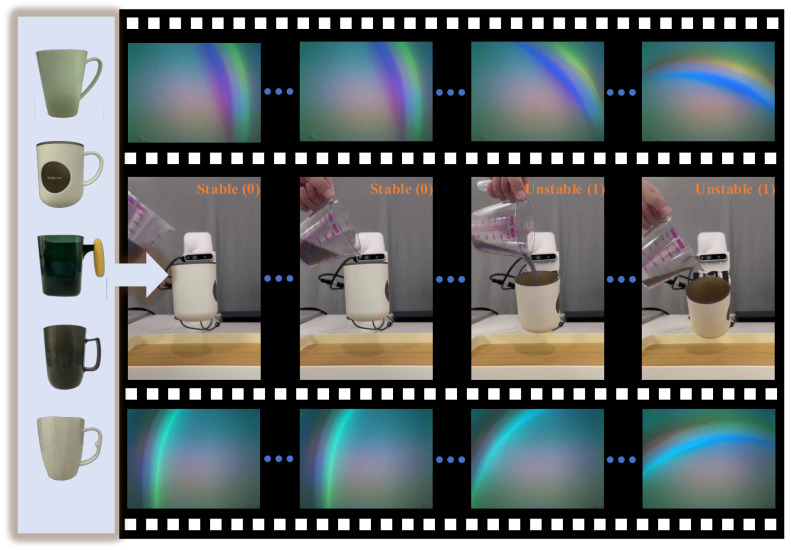
Data collection. Five cups with distinct handles are employed to collect tactile data, incorporating varied grasp forces corresponding to each handle. It is observed that several factors contribute to grasp stability, encompassing the grasping position, applied force, and fluctuations in the object’s weight. Additionally, a notable trend emerged during image acquisition from tactile sensors, revealing rotational occurrences in the detection of object features.

**Figure 5:**
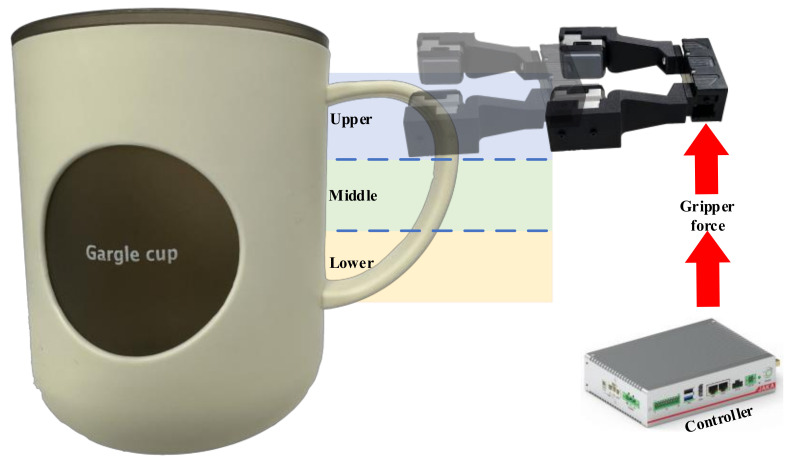
Grasping configuration. We partition the cup’s handle into three sections: upper, middle, and lower. Subsequently, the gripper applies different grasping forces (15 N, 25 N, 40 N, 50 N) to grasp each of the three parts separately.

As shown in Figure 4, there are five different cups, each featuring a unique handle design. We categorize the handle into three segments: upper, middle, and lower. Employing the gripper, we grasp various sections of the cup handle while maintaining an equal distribution ratio of 1:1:1 (see Figure 5). The gripper force is adjustable, and we configure four different force levels: 30%, 50%, 80%, and 100% of the gripper’s maximum capacity (15 N, 25 N, 40 N, 50 N). The interaction duration with humans is limited to 6 s, aligning with the acquisition time for each video from tactile sensors. Operating at a frequency of 60 Hz, each video comprises 360 frames. We obtain a dataset of 21,600 frames from a total of 60 videos, each possessing a spatial resolution of 320 × 240 pixels. For model training, 48 videos are utilized, while the remaining 12 videos are reserved for testing. Notably, we categorize these videos based on the objects being grasped, ensuring that each object appears exclusively in either the training or testing dataset. Finally, every frame is labeled as either a stable (0) or unstable (1) grasp, maintaining a balanced ratio of stable and unstable instances to mitigate class imbalance. We define the stability of grasp as follows: if the tactile images do not change throughout the video, we label each frame as stable (0) and consider the object to be stably grasped. If the tactile image changes compared to the first frame during the video, indicating that the object is unstably grasped, we label that frame as unstable (1).

## 4. Methodology

**Figure 6:**
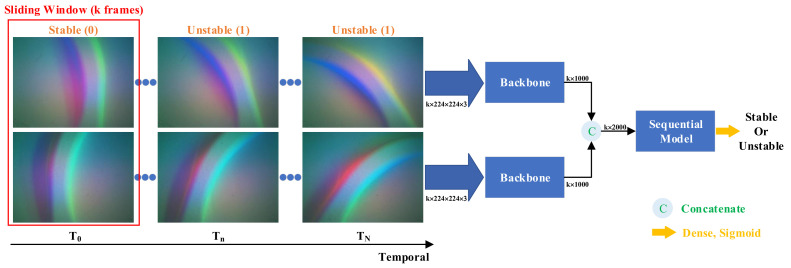
Overview of network framework. The temporal sequences from the left and right tactile sensors (Gelsight Mini) serve as dual inputs for the classification network. Employing a sliding window of size k frames, we traverse the tactile temporal sequences. Consequently, the input shape of the network is defined as (k, height, width, channel), with k denoting the number of timesteps. The corresponding label for each input is established by determining the maximum label value within the k frames. We utilize pretrained models such as ResNet50 [48], ResNet101 [48], DenseNet121 [49], etc., as the backbone for the network framework. We feed the output of the backbone into sequential models, such as LSTM [54] or Transformer [55], to handle temporal sequences. However, the final choice is contingent upon assessing their classification accuracy, allowing us to determine the most suitable model for our specific application.

As shown in Figure 6, the design of the network framework takes into account the characteristics of the collected dataset and aligns with the objectives of the task. The framework primarily comprises two main components: convolutional neural networks (CNNs) [53], serving as the backbone for spatial feature extraction, and a sequential model designed to capture temporal information.

### 4.1. Convolutional Neural Networks (CNNs)

Convolutional neural networks (CNNs) are specialized neural networks designed for processing data with spatial relationships, and widely applied across domains such as image processing and time series prediction [56]. Their framework mainly includes three parts: an input layer, an output layer, and multiple hidden layers. These hidden layers contain convolutional layers that perform dot products between the input matrix and the convolution kernel [57]. Considering the characteristics of our dataset, we will employ a CNN framework to extract spatial features from the video sequences.

In the illustration of Section 3.2, each video is constrained to a duration of 6 s, comprising a total of 360 frames. To input the network framework and utilize temporal information, we employ a sliding window to sample the video (see Figure 6). Each frame, denoted as **I**(T_*n*_), is labeled either 0 or 1, representing stable or unstable, respectively. The corresponding label G(T_*n*_) is determined as the maximum value within the sequence [G(T_*n*_), G($T_{n+1}$), ⋯, G($T_{n+k-1}$)]. We feed the temporal sequences with a shape of (k, weight, height, channel) into distinct upper and lower channels of the network framework. k denotes the number of timesteps. Following that, we utilize pre-trained ImageNet models as the backbone to extract spatio features from these sequences. For instance, if we opt for ResNet50 [48] as the backbone, we retain its fully connected layer, resulting in an output shape of (k, 1000).

We then concatenate these two outputs to yield a final output shape of (k, 2000). To maintain the timesteps dimension of the backbone module and the concatenated layer, we employ the TimeDistributed layer (https://keras.io/api/layers/recurrent_layers/time_distributed/ (accessed on 19 July 2024)), a valuable tool for handling time series data or video frames. This layer enables the application of a single model to each input, simplifying the management of data over time. Finally, the output, shaped as (k, 2000), is fed into a sequential model. LSTM [54] and Transformer [55] are widely recognized as popular sequential models, as detailed in the following section.

### 4.2. Sequential Models

#### 4.2.1. Long Short-Term Memory (LSTM)

Long short-term memory (LSTM) is a specialized form of recurrent neural network (RNN) [54]. LSTM is adept at processing sequential data by retaining a memory of past inputs. Unlike conventional feed-forward neural networks that analyze data in a single pass, LSTM is tailored to manage data with temporal dependencies, such as time series. The LSTM cell has several key components:

(1) Forget Gate $f_{t}$

(1) $$f_{t}=\sigma \left(W_{f}\cdot \left[h_{t-1},x_{t}\right]+b_{f}\right)$$

(2) Input Gate $i_{t}$

(2) $$i_{t}=\sigma \left(W_{i}\cdot \left[h_{t-1},x_{t}\right]+b_{i}\right)$$

(3) Output Gate $o_{t}$

(3) $$o_{t}=\sigma \left(W_{o}\cdot \left[h_{t-1},x_{t}\right]+b_{o}\right)$$

where σ represents the sigmoid activation function, $\left[h_{t-1},x_{t}\right]$ denotes the concatenation of the previous hidden state $h_{t-1}$ and the current input $x_{t}$, and $W_{f}$, $W_{i}$, $W_{o}$ are weight matrices, while $b_{f}$, $b_{i}$, $b_{o}$ are bias vectors. The Forget Gate $f_{t}$ decides which information from the cell state $c_{t-1}$ should be discarded. The Input Gate $i_{t}$ determines which new information from ${\tilde{c}}_{t}$ (Equation (6)) should be stored in the cell state. The Output Gate $o_{t}$ regulates the information that will be output as the hidden state $h_{t}$ (Equation (4)).

(4) $$h_{t}=o_{t}\cdot \tanh\left(c_{t}\right)$$

(5) $$c_{t}=f_{t}\cdot c_{t-1}+i_{t}\cdot {\tilde{c}}_{t}$$

(6) $${\tilde{c}}_{t}=\tanh\left(W_{c}\cdot \left[h_{t-1},x_{t}\right]+b_{c}\right)$$

where $W_{c}$ is a weight matrix, $b_{c}$ is a bias vector.

Therefore, LSTM is designed to selectively remember or forget information over long sequences, making it effective for capturing dependencies in time series or sequential data.

#### 4.2.2. Transformer

Transformer is a neural network architecture based on attention mechanisms. Its strength lies in efficiently processing data with temporal information, especially in the context of time series data, by capturing the relationships across different positions in the sequence through global attention. The Transformer consists of several essential components: self-attention mechanism, multi-head attention, and positional encoding.

The self-attention mechanism computes a set of attention scores for each element in the input sequence. The attention scores are used to form a weighted sum, allowing the model to focus on different parts of the input sequence differently.

(7) $$\mathrm{Attention}\left(Q,K,V\right)=\mathrm{softmax}\left(\frac{QK^{T}}{\sqrt{d_{k}}}\right)V$$

where *Q*, *K*, and *V* represent the query, key, and value matrices, respectively. $d_{k}$ is the dimensionality of the key vectors.

To enhance the model’s ability to capture diverse patterns, multiple self-attention mechanisms, or attention heads, are employed in parallel.

(8) $$\mathrm{MultiHead}\left(Q,K,V\right)=\mathrm{Concat}\left({\mathrm{head}}_{1},\ldots ,{\mathrm{head}}_{h}\right)W_{O}$$

where *h* is the number of heads, and $W_{O}$ is the output matrix.

Since Transformer lacks inherent sequential order information, positional encodings (PosE) are added to the input embeddings to impart knowledge of the position of elements in the sequence. Two separate formulas are employed for encoding positional information along both the even and odd dimensions. This is to ensure that the model can distinguish between different positions effectively.

Even dimensions (2γ):

(9) $$\mathrm{PosE}\left(pos,2\gamma \right)=\sin\left(\frac{pos}{10000^{2\gamma /d_{\mathrm{model}}}}\right)$$

Odd dimensions (2γ + 1):

(10) $$\mathrm{PosE}\left(pos,2\gamma +1\right)=\cos\left(\frac{pos}{10000^{2\gamma /d_{\mathrm{model}}}}\right)$$

where pos represents the position of the element in the sequence, γ represents the dimension index, and $d_{model}$ is the dimensionality of the model.

These components collectively enable the Transformer to effectively model and process sequential data, offering significant advantages in various applications.

As previously mentioned, we leverage both LSTM and Transformer architectures to manage temporal data. Finally, the output from the LSTM or Transformer is directed into a dense layer featuring a sigmoid activation function, culminating in the generation of prediction results. The ultimate selection between LSTM and Transformer hinges on an evaluation of classification accuracy, enabling us to identify the most fitting model for our particular application.

Our method is designed to be both predictive and proactive in nature. By continuously monitoring the tactile feedback, the system is capable of detecting early signs of instability. This approach allows the system to provide an early warning and trigger corrective actions before significant instability occurs. Specifically, our method analyzes subtle changes in the tactile images to anticipate potential issues and maintain a stable grasp proactively.

## 5. Experiment and Results

### 5.1. Implementation and Experimental Setup

We conduct our experiments using Keras/TensorFlow on NVIDIA GeForce RTX 4090 GPU servers. The binary crossentropy of Keras serves as the loss function for the entire network, predicting a probability distribution over classes through a sigmoid function. For optimization, we employ the Adam optimizer [58] with parameters (batchsize = 4, β1 = 0.9, β2 = 0.999, ε = 0.001, learning rate = 0.001), without incorporating learning rate decay. The network is trained for 100 epochs based on the collected dataset. A preprocessing step is applied to the videos, resizing each frame to an image size of 224 × 224 pixels, which aligns with the input shape requirements of pretrained ImageNet models.

We evaluate the performance of our method using classification accuracy as the metric. This measure is determined by the ratio of the number of correct predictions to the total number of predictions made. The emphasis on maintaining a balanced distribution of data during the collection process contributes to achieving a high classification accuracy, showcasing the effectiveness of both the proposed classification network and the collected dataset.

### 5.2. Results and Discussion

In accordance with Figure 6, there is a need to define specific hyperparameters and network structures. Given a sliding window size of k = 8, our subsequent experiments involve testing different pretrained ImageNet models to identify the most optimal backbone for our framework. In Table 1, we evaluate various pretrained models as backbones in conjunction with LSTM [54] and Transformer [55]. Comparative analysis with EfficientNetB0 [59], ResNet50 [48], and ResNet101 [48] reveals that the highest classification accuracy is consistently achieved when DenseNet121 [49] is employed as the backbone based on LSTM, reaching an accuracy of 98.90%. Therefore, we choose DenseNet121 as the backbone, ensuring efficient classification without imposing a significant computational burden. This choice enables real-time feedback, with an inference time of 31.84 ms during the human–robot interaction stage. The inference time of 31.84 ms refers to the time it takes for our algorithm to process an entire video and produce a stability prediction, which includes the time required for feature extraction, running the model, and outputting the stability status.

**Figure 7:**
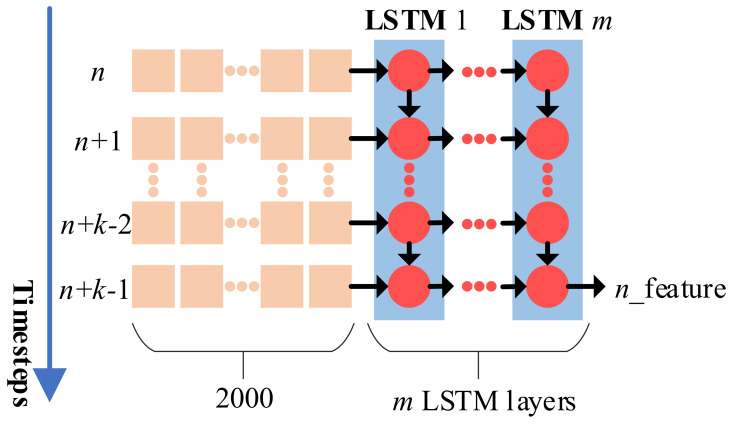
*m* LSTM layers. In the sequential model of Figure 6, the composition of LSTM layers varies. When a single LSTM layer is employed, its output has a shape of [n_feature]. However, if multiple LSTM layers are utilized, the final layer retains the shape [n_feature], while the output of preceding LSTM layers takes the form [*k*, n_feature]. This is because the outputs of the additional LSTM layers encompass all hidden states across each time step.

In Table 1, we exclusively employ a single LSTM layer. To explore the influence of the composition of LSTM layers, we conduct tests with varying numbers of LSTM layers (see Figure 7). As the number of LSTM layers increases, there is a noticeable reduction in classification accuracy (see Table 2). This observation suggests that an indiscriminate increase in the number of layers may not necessarily lead to improved classification accuracy. The decrease in accuracy when using more than one LSTM layer can be attributed to overfitting due to the increased model complexity, the vanishing gradient problem during training, and the relatively small size of our dataset (60 videos), which is insufficient to support deeper networks. Additionally, increased computational complexity with more layers can lead to longer training times and suboptimal convergence. Hence, we ultimately configure the LSTM with a single layer in the network framework.

The aforementioned experiments primarily revolve around a sliding window size of k = 8. However, the performance of the classification model is influenced by the choice of sliding window size. Consequently, we proceed to assess the impact of various sliding window sizes. In Table 3, the classification accuracy is presented for sliding window sizes ranging from 2 to 8. The optimal result is achieved with a sliding window size of 8. Setting the sliding window size too small or too large does not yield optimal classification accuracy for the model.

**Figure 8:**
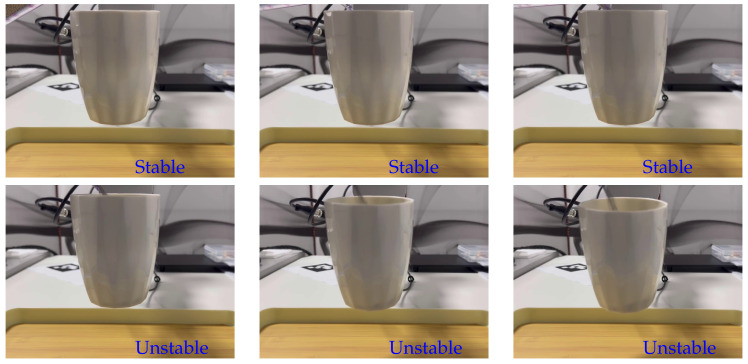
Grasp stability prediction on the robotic platform. The two-jaw parallel grippers, equipped with GelSight tactile sensors, randomly grasp the cup’s handle, while the cup’s weight undergoes continuous changes during interactions with humans.

To validate the efficacy of implementing our proposed method on a real robot platform, we continuously output the grasp state (stable/unstable), as illustrated in Figure 8. Our experimental results demonstrate that the proposed method effectively provides early warnings of potential instability. By detecting subtle changes in tactile feedback before significant instability manifests, the method allows for proactive adjustments to be made, thereby maintaining a stable grasp.

In this study, our method offers several advantages over existing methods that measure grasp slip or stability:

(1) Unlike traditional incipient slip detection methods that react to the onset of slip, as shown in the grasping object phase of Figure 1, our approach continuously monitors tactile feedback to detect both subtle and significant changes in the human–robot interaction phase of Figure 1. This allows for early detection and proactive adjustments. As shown in Figure 8, this continuous monitoring successfully identified instability before any significant slippage occurred, showcasing the method’s effectiveness in early detection.
(2) Our method is designed to provide real-time feedback, predicting potential instability before it fully manifests. This early warning system enables corrective actions to be taken proactively, which is crucial in dynamic human–robot interaction scenarios. In our results (Table 1 and Table 2), we observed that our method could detect instability transitions with an average inference time of 31.84 ms per video.
(3) Our method’s inference time is of 31.84 ms per video, which ensures rapid response to potential instability. The continuous monitoring and comparison of tactile images ensure high accuracy in detecting changes in grasp stability. Specifically, our method achieved an accuracy rate of 98.9% in detecting instability transitions. Although a direct quantitative comparison with other methods in terms of speed and accuracy was not performed in this study, our results demonstrate that the proposed method can effectively avoid unstable grasps.

Although we propose that our method can offer real-time feedback to improve human–robot interaction, the present study does not include experiments wherein such feedback is provided to humans. Consequently, while our results demonstrate the method’s capability to detect instability, in the future, further research is necessary to validate the effectiveness of real-time feedback in enhancing human–robot interaction.

## 6. Conclusions

In this paper, we introduce a real-time dynamic state sensing network that combines DenseNet121 [49] and LSTM [54] to predict changes in the robot’s state during human–robot interaction. Our approach begins with the creation of a tactile sensing dataset, recorded during the interaction between humans and the robot, serving as a fundamental component for data-driven methods. To leverage temporal information, we employ a sliding window with a size of 8 to sample the obtained videos, feeding them into the classification network for real-time feedback on the robot’s state changes, enabling humans to respond appropriately. Additionally, we validate the model’s generalization by applying it to unseen objects, achieving an average classification accuracy of 98.90%. In the future, our focus will be on providing corrective actions to enhance the smooth and effective interaction between humans and robots. We plan to develop a fully closed-loop control system that enables the robot to adeptly navigate the transition from a stable state to an unstable state.

## Figures and Tables

**Table 1:** Classification accuracy percentage of different backbones on the test dataset.

	Backbone	Accuracy (%) ↑	Parameters ↓	Time (ms) per Inference Step ↓	Size (MB) ↓
LSTM [54]	DenseNet121 [49]	**98.90**	8,126,801	31.84	62.72
	EfficientNetB0 [59]	92.27	5,394,868	22.32	41.43
	ResNet50 [48]	96.13	25,701,009	25.81	196.3
	ResNet101 [48]	95.58	44,771,473	42.61	342.22
Transformer [55]	DenseNet121 [49]	96.69	9,242,921	33.56	66.99
	EfficientNetB0 [59]	91.71	6,510,988	23.84	45.71
	ResNet50 [48]	90.61	26,817,129	26.78	200.58
	ResNet101 [48]	87.29	45,887,593	44.96	346.5

Note: LSTM [54] is configured with a single layer in the network framework of Figure 6, and parameters have an output space dimensionality of 8, returning the last output in the output sequence. For Transformer [55], a single layer is utilized, focusing solely on its encoder module. The parameters are set with 4 attention heads, each with a size of 32 for both query and key. The bold formatting indicates the best accuracy.

**Table 2:** Classification accuracy percentage of varying numbers *m* of LSTM [54] layers on the test dataset.

*m*	Accuracy (%) ↑	Parameters ↓	Time (ms) per Inference Step ↓	Size (MB) ↓
1	**98.90**	8,126,801	31.84	62.72
2	97.24	8,127,345	36.00	62.72
4	95.03	8,128,433	37.72	62.74

Note: The bold formatting indicates the best accuracy.

**Table 3:** Classification accuracy percentage with varying numbers *k* of sliding windows on the test dataset.

*k*	Accuracy (%) ↑
2	88.15
3	94.66
4	94.03
5	94.39
6	94.76
7	96.77
8	**98.90**
9	95.45
10	96.49
11	95.78

Note: The bold formatting indicates the best accuracy.

## Data Availability

Data are contained within the article.
